# Third Dentition

**Published:** 1869-05

**Authors:** J. K. Dodge

**Affiliations:** West Eau Claire, Wis.


THIRD DENTITION—By J. K. DODGE, West Eau Claire, Wis.—Two cases of third dentition have lately come to my notice, which may be of interest to the profession. One was that of a lady of 48 years, who had her teeth taken out eight months since, and who now has a right superior cuspid erupting, and I think there are signs of others. The second case was that of a daughter of the first, aged 18, who had her teeth taken out some six months since; a right superior cuspid appeared, which I extracted. These are interesting cases, happening the same to both mother and daughter.—*Dental Cosmos.*

THIRD DENTITION.—Dr. H. H. Nelles, Dentist, reports in the *Canada Journal of Dental Science*, the case of a lady forty-five years of age, from whom he extracted a number of roots preparatory to the insertion of a full set of artificial dentures. Upon a second visit, to his surprise, he found that nature had sent forth a well-developed superior *cuspidatus*. In anticipation that nature would complete the work thus auspiciously begun, Dr. Nelles deferred inserting an artificial set of teeth. This case brings to mind a fact related of himself, by a gentleman of our acquaintance. When he was 19 years of age, his four upper incisors were broken off by a blow with an iron bar. The roots were extracted, but no artificial teeth inserted. Some months after, while at the table eating, he was greatly startled to discover the points of teeth projecting through the gum. The incisors soon fully supplied the place of the lost second teeth, and are as perfect in every particular as one could wish.—HENRY GIBBONS, Jr., M. D. *Pacific Med. & Surg. Jour.*

